# A survey of chlorhexidine oral care in scottish intensive care units

**DOI:** 10.1186/2197-425X-3-S1-A710

**Published:** 2015-10-01

**Authors:** J Small, R Campbell, L Paton, A Mackay

**Affiliations:** Victoria Infirmary/ NHS Greater Glasgow & Clyde, Glasgow, United Kingdom

## Introduction

Daily oral care with chlorhexidine has been widely recommended as part of care bundles for ventilated patients by organisations such as the Scottish Patient Safety Programme (SPSP) [1], and the Institute for Healthcare Improvement (IHI) despite studies showing variable efficacy.

## Objectives

A recent systemic review [2] and network meta-analysis suggested that chlorhexidine used for mouth care is associated with an increase in mortality. We therefore developed a survey to quantify the use of chlorhexidine in Scottish intensive care units.

## Methods

On a single day, we called each of the 24 intensive care units in Scotland and spoke to the Charge Nurse on duty. We asked them the following questions using a standard survey form:

1. Have you ever given chlorhexidine mouth care as routine to all intubated patients?

2. Do you currently give chlorhexidine mouth care as routine to all intubated patients?

3. If you stopped, when and why?

4. Are you reviewing your practice in light of recent evidence suggesting potential harm associated?

Where appropriate, the opportunity was taken to spread awareness of the recently published evidence.

## Results

Twenty four (100%) of units have used chlorhexidine mouth care at some stage.

Four out of 24 (17%) units no longer use chlorhexidine. All of these units stopped as a direct result of the recent meta-analysis.

Of the units still using chlorhexidine, 3 (13%) claimed to be aware of the meta-analysis and one of these units was currently reviewing their practise.

## Conclusions

Chlorhexidine oral care has been a staple of ICU care since the introduction of standardised bundles of care by the SPSP. Our unit felt that the lack of clear evidence of benefit combined with the recent evidence of excess mortality justified discontinuing its recent use.

In an era of evidence based medicine, it is interesting to see the rate at which best evidence is adopted, and to this end we plan on repeating our survey after 12 months to assess the impact of this important study.
